# Unnatural Deaths in Shanghai from 2000 to 2009: A Retrospective Study of Forensic Autopsy Cases at the Shanghai Public Security Bureau

**DOI:** 10.1371/journal.pone.0131309

**Published:** 2015-06-25

**Authors:** Meng He, You-Xin Fang, Jun-Yi Lin, Kai-Jun Ma, Bei-Xu Li

**Affiliations:** 1 Department of Forensic Medicine, School of Basic Medical Sciences, Fudan University, Shanghai 200032, P. R. China; 2 Department of Neurology, Huashan Hospital, Fudan University, Shanghai 200040, P. R. China; 3 Shanghai Key Laboratory of Crime Scene Evidence, Institute of Forensic Science, Shanghai Public Security Bureau, Shanghai 200083, P. R. China; Xi'an Jiaotong University School of Medicine, CHINA

## Abstract

Shanghai is the most developed city in China and has a soaring population. This study uses forensic epidemiology to determine the relationship between unnatural deaths and the development in Shanghai, based on recently released forensic autopsy cases from the 2000s at the Shanghai Public Security Bureau (SPSB). There were 5425 accidental deaths, 2696 homicides, 429 suicides, 186 natural deaths, and 1399 deaths of undetermined cause. There was a male-to-female ratio of 2.02:1, and the average age was 40.9±18.7 years. Traffic accidents (84.2%) were the number one cause of accidental deaths, which decreased during the study period. Sharp force injury (50.6%) was the leading cause of homicides, different from Western countries, where firearms are the leading cause. Hanging (24.5%) was the leading cause of suicides, whereas drug and chemical intoxication was the leading cause in the previous decade; pesticide ingestion decreased in the 2000s. In addition to traffic accidents, manual strangulation was the leading cause of death in childhood fatalities. Children under age 2 were vulnerable to homicides. In the 2000s, there were a large number of drug overdoses, and illegal medical practices and subway-related deaths first appeared in Shanghai. A new type of terrorist attack that involved injecting people with syringes in public places was reflected in the SPSB archives. The forensic epidemiology and changes in unnatural deaths in this decade reflected their relationship with the law, policy and changes in Shanghai. Illegal medical practices, subway-related deaths and terrorist attacks were closely related to the development in Shanghai. Identifying the risks of unnatural deaths will improve public health.

## Introduction

Shanghai, with a population over 24 million, is one of the largest cities in China, and the progress in globalization was rapid in the 2000s. The city, located in the Yangtze River Delta, is seen as China’s commerce, finance and science center; it is a typical developed Chinese city.

The Shanghai Public Security Bureau (SPSB) is responsible for public security, crime control, and traffic administration. The SPSB has its own medical examiners (MEs), whose duty is to support the Bureau in forensic investigation. In addition, there are independent, non-SPSB MEs affiliated with university forensic medicine departments, private forensic science centers, and the Ministry of Justice Institute of Forensic Science. The procedure for investigating unnatural deaths in Shanghai is as follows. First, the death is initially reported to the SPSB. Second, the police, forensic investigators and SPSB MEs arrive at the scene, conduct the primary examination and collect evidence. Third, based on the scene investigation and initial corpse inspection, if there are indications of foul play or suspicious activity, a complete forensic autopsy is performed by SPSB MEs and an additional investigation is conducted. If the death investigation and scene inspection reveal no evidence of suspicious activity or trauma, no autopsy is performed, and the SPSB closes the case. In addition, accidental traffic fatalities are managed by the SPSB’s traffic police, who are relatively independent within the Bureau and who manage the city’s traffic. Accidental traffic fatalities are initially reported to the SPSB traffic police, and the victim is inspected by SPSB MEs or by an independent third party.

We could not obtain earlier forensic autopsy case data because of the SPSB’s previous confidentiality policies, but data from the 2000s were recently released. As such, we can conduct a retrospective study of unnatural deaths in Shanghai from 2000 to 2009.

## Materials and Methods

This study was a retrospective review of unnatural deaths at the SPSB over a 10-year period from 2000.1 to 2009.12. Cases were accepted for this study according to the following criteria: (1) corpses without death certificates were found in Shanghai; (2) corpse inspection was conducted by SPSB MEs; (3) manner of death was given including undetermined. A total of 10135 cases met the study criteria. These archives were kept in the SPSB records office.

Data were extracted from these cases’ SPSB autopsy archives, the initial investigations, and police reports. These materials were reviewed and analyzed as to (1) victim demographic data, such as age and gender; (2) manner of death; (3) cause of death; (4) decedents ≤16 years; (5) drug abuse deaths; (6) illegal medical practice; and (6) subway-related deaths. Two reviewers independently extracted data from each of the cases. Disagreements between reviewers were resolved through discussions and a final consensus.

The data were presented as the means-standard errors (SE) for age, and the Student t-test was used to compare the ages in the different groups. The statistical analysis was performed with Microsoft Excel 2013 and SPSS 12.0, and the differences were considered significant when p was less than 0.05. This retrospective study was based on the forensic autopsy case archives. It was approved by both the Shanghai Public Security Bureau Ethics Committee and the Fudan University Ethics Committee. Written informed consent was given by the decedents’ next of kin, who were told at the time of the autopsy that the investigation information might be used in scientific research. All cases were anonymized and de-identified prior to analysis.

## Results

**Fig 1:**
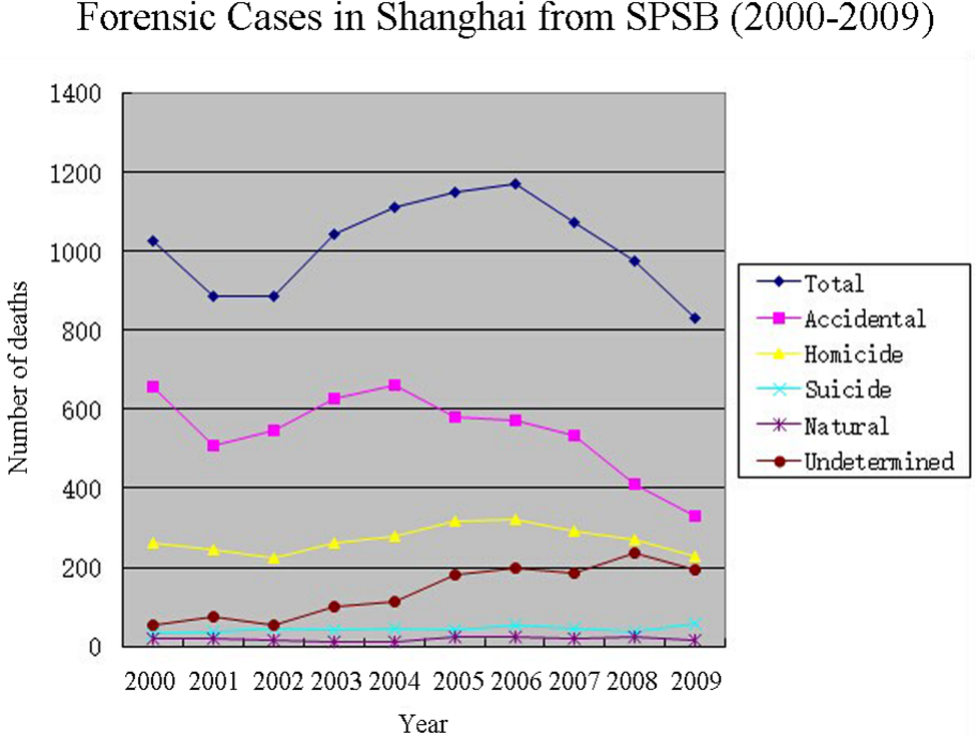
Forensic Cases in Shanghai from SPSB (2000–2009).

Of the 10135 decedents, 6717 were male and 3327 were female, a male-to-female ratio of 2.02:1, excluding 91 decedents whose genders were unknown or unrecorded. The average age of decedents in this decade was 40.9±18.7 years. Table 1 and Fig 1 show that the total number of forensic cases investigated by the SPSB fluctuated throughout the decade, with a minimum of 822 in 2009 and a maximum of 1170 in 2006. Accidents and homicides followed the same trend as the total forensic cases, successively decreasing, increasing and decreasing. Suicides and deaths from natural causes remained relatively stable, approximately 42 and 18 per year, respectively. Undetermined cases increased from 55 to 236, with a slight decrease to 196 in 2009. Between 2000 and 2009, SPSB data revealed that there were 5425 accidental deaths, 2696 homicides, 429 suicides, 186 natural deaths, and 1399 deaths of undetermined cause.

**Table 1:** All Deaths from SPSB’s Forensic Archives (2000–2009).

Year	Traffic Accidents	Non-traffic Accidents	Homicides	Suicides	Natural	Undetermined	Total
2000	461	195	264	32	18	55	1025
2001	400	109	244	36	20	76	885
2002	481	65	223	44	16	55	884
2003	529	98	261	42	10	101	1041
2004	577	84	278	45	13	114	1111
2005	521	61	319	41	25	182	1149
2006	507	66	320	52	26	199	1170
2007	462	71	292	45	18	185	1073
2008	360	50	269	36	24	236	975
2009	269	59	226	56	16	196	822
Total	4567	858	2696	429	186	1399	10135

### Accidents

**Fig 2:**
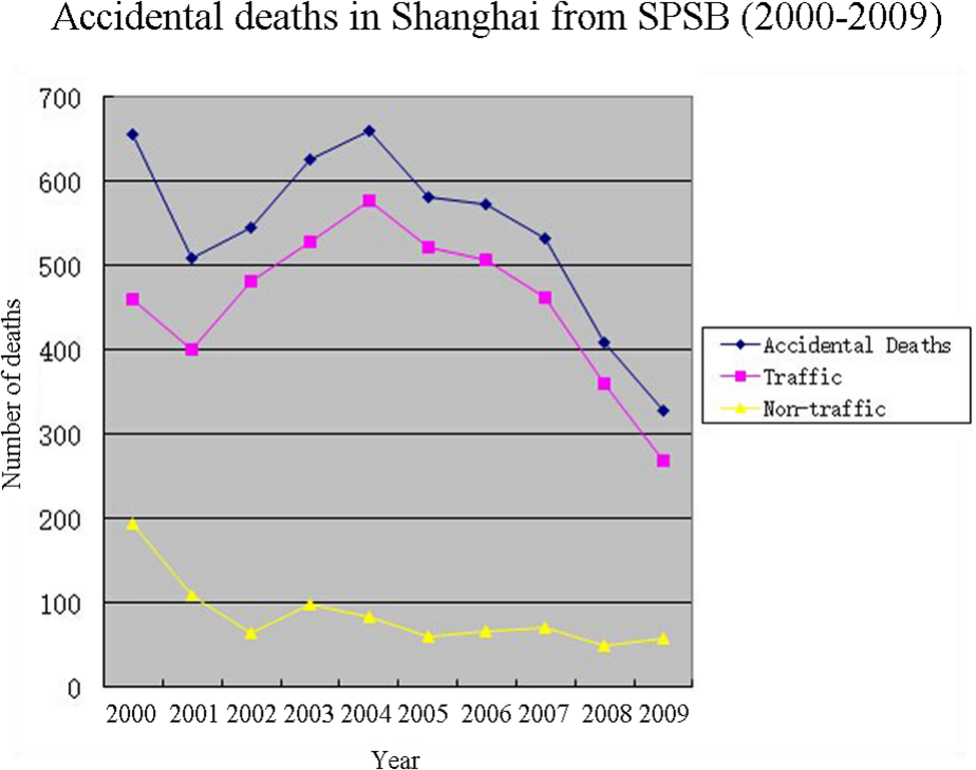
Accidental Deaths in Shanghai from SPSB (2000–2009).

Based on practice and jurisdiction, accidental deaths were divided into traffic and non-traffic. Non-traffic accidents referred to accidents that typically occurred at work, especially at construction sites. The majority of accidental deaths (84.2%) were traffic fatalities, which also comprised the largest proportion of all SPSB forensic cases (45.0%, 4567/10135). Traffic fatalities fell from 461 to 400 in 2001 and rose quickly to 577 over the next three years; they ultimate fell sharply, to 269, in 2009 (see Fig 2). The male-to-female traffic accident ratio was 2.31: 1. The average age of traffic accident victims was 44.9±19.2 years. The changes in traffic fatalities contributed to the trend in total accidental deaths as well as the trends in the total number of forensic cases.

There were considerably fewer accidental non-traffic than traffic fatalities. The number of non-traffic accidental deaths decreased from 195 to 65 in the first three years and remained at approximately 70 per year between 2003 and 2009. The male-to-female non-traffic accident ratio was 2.23: 1. The average age of non-traffic accident victims was 37.7±19.0 years.

Table 2 shows the causes of accidental deaths, with the leading causes as follows: traffic fatalities (84.2%), non-traffic fatalities (6.3%), drowning (1.7%), carbon monoxide intoxication (1.5%), thermal injuries (burns) (1.4%), and mechanical asphyxia (1.0%). Accidental non-traffic fatalities included: (1) sharp force injuries caused by sharp equipment such as propellers, cutting machines, and glass; (2) blunt force injuries caused by blunt equipment impacts, such as falling rocks, pile drivers, and elevators; and (3) large-scale mechanical equipment accidents, such as slipping and falling into a blender. Forty-six cases (0.8%) had no recorded cause of death. Cause of death was listed as infections, hemolysis, or shock in 13 cases (0.2%).

**Table 2:** Causes of Accidental Deaths in Shanghai (2000–2009).

Cause of death	Number	%
Traffic accidents	4567	84.2
Non-traffic accidents *	340	6.3
Drowning	90	1.7
Carbon monoxide intoxication	84	1.5
Thermal injuries (burns)	74	1.4
Mechanical asphyxia	55	1.0
Falls from heights	49	0.9
Electrocution	42	0.8
Drug abuse	26	0.5
Intoxication (unknown or unrecorded drug or chemical)	21	0.4
Hypothermia or starvation	6	0.1
Alcohol intoxication	5	0.09
Nitrite intoxication	2	0.04
Tetrodotoxin intoxication	2	0.04
Gunshot wound	2	0.04
Tetramine intoxication	1	0.02
Others **	13	0.2
Unknown or unrecorded	46	0.8
Total	5425	100

* Non-traffic fatalities referred to those caused by: (1) sharp force injuries from sharp equipment, such as propellers, cutting machines, glass, etc.; (2) blunt force injuries from blunt equipment impact, such as falling rocks, pile drivers, and elevators; (3) and large-scale mechanical equipment accidents such as slipping and falling into a blender.

** Causes of death were listed as hemorrhagic shock, infection, or hemolysis, but the underlying causes were not recorded and could not be determined.

### Homicides

A total of 2696 homicides occurred in Shanghai from 2000 to 2009. The annual number of homicide victims decreased from 264 to 223 in 2002 and then increased to 320 over the next four years, finally decreasing to 226 in 2009 (see Fig 1). The male-to-female homicide ratio was 1.47: 1. The average age of homicide victims was 36.2±15.9 years. Table 3 shows the causes of homicidal deaths, with the five leading causes being: sharp force injuries (50.6%), blunt force injuries (23.1%), ligature and manual strangulation (17.2%), smothering (1.8%), and drowning (1.0%). There were only 14 gunshot victims (0.5%), and six victims of illegal medical practices were recorded in the SPSB archives. Of the 2696 homicide cases, 91 (3.4%) had no listed cause of death.

**Table 3:** Causes of Death in Homicides, Shanghai (2000–2009).

Cause of death	Number	%
Sharp force injuries	1367	50.7
Blunt force injuries	624	23.1
Strangulation	465	17.2
Smothering	49	1.8
Drowning	26	1.0
Carbon monoxide intoxication	19	0.7
Gunshot wound	14	0.5
Thermal injuries (burns)	10	0.4
Illegal medical practice	6	0.2
Intoxication (unknown or unrecorded drugs or chemicals)	5	0.2
Injuries due to fall from height	5	0.2
Electrocution	4	0.1
Tetramine intoxication	4	0.1
Prescription drugs intoxication	1	0.04
Explosion injury	1	0.04
Cyanide intoxication	1	0.04
Others *	4	0.1
Unknown or unrecorded	91	3.4
Total	2696	100

* Causes of death were listed as infection, diffuse peritonitis or septicemia, or shock, but the underlying causes were not recorded and could not be identified.

Notably, 7 syringe-injection cases, which were considered terrorist attacks, occurred in September and October 2009. These victims were injected with syringes that were claimed to contain HIV-infected blood. The victims did not die, and they were not infected with HIV, and HIV was not detected in any of the blood as well; thus, syringe injections were not a cause of death. However, these attacks were included in SPSB’s homicide archives because the cases were very special and required extra resources for investigation.

### Suicides

Among the suicide victims, the male-to-female ratio was 2.08:1, and the average age was 38.5±14.8 years. Different from homicide, the five leading causes of death in the suicides were: hanging (24.5%), jumping from heights (24.0%), drug or chemical intoxication (20.5%), drowning (10.7%), and cutting and stabbing (10.3%). Of the 88 intoxication cases, carbon monoxide was the most common agent used by the victims (34/88), followed by ingesting pesticides such as organophosphorus (22/88). There were 25 cases with cause of death listed as intoxication by unknown drug or chemical (Table 4).

**Table 4:** Causes of Death in Suicides, Shanghai (2000–2009).

Cause of death	Number	%
Hanging	105	24.5
Jumping from heights	103	24.0
Drug or chemical intoxication (Carbon monoxide)	34	7.9
Drug or chemical intoxication (Pesticides)	22	5.1
Drug or chemical intoxication (Tetramine)	3	0.7
Drug or chemical intoxication (Prescription drugs)	2	0.5
Drug or chemical intoxication (Illicit drugs)	2	0.5
Drug or chemical intoxication (Unknown or unrecorded)	25	5.8
Drowning	46	10.7
Cutting and stabbing	44	10.3
Multiple injuries	19	4.4
Thermal injuries (burns)	10	2.3
Explosion injury	4	0.9
Electrocution	3	0.7
Gunshot wounds	3	0.7
Unknown or unrecorded	4	0.9
Total	429	100

### Childhood Fatalities [1]

All childhood fatality cases were drawn from the aforementioned cases. A total of 491 deaths of children age ≤16 years were investigated by the SPSB in Shanghai during the 2000s. The ages ranged from newborn to 16 years, with an average age of 7.2±5.7 and a male-to-female ratio of 1.36:1. For the 491 children, the manner of death was: accident, 278 cases; homicide, 108 cases; suicide, 5 cases; natural, 12 cases; and undetermined, 88 cases. Of the 108 homicide victims (60 male, 48 female), the leading cause of death was manual strangulation. Eighteen of the 108 victims (16.7%) were under 2 years of age, and of these 18, more victims were female than male, at a ratio of 11:7.

### Drug Abuse Deaths

All of the drug abuse cases were drawn from the aforementioned cases. From 2000 to 2009, a total of 38 drug abuse deaths were recorded. For the drug abuse deaths, the male-to-female ratio was 1.24:1, and the average age was 29.4±9.0 years; these decedents were markedly younger than all others (p<0.05). Of the 38 deaths, the manners of death were accident (26 cases), suicide (2 cases), and undetermined (10 cases).

### Illegal Medical Practices

All of the illegal medical practice cases were drawn from the aforementioned homicide cases. In the decade of study, 6 deaths from illegal medical practices were recorded, with 4 deaths related to childbirth. Among these, 1 decedent was an infant who had died from dystocia, and 3 decedents were pregnant women, average age 30.7 years, who died from blood loss during delivery. In addition, the exact cause of death was unrecorded for 1 female and 1 male.

### Subway-related Deaths

All of the subway-related deaths were drawn from the aforementioned cases. In this decade, 6 deaths from subway train impact were recorded. All decedents were males, with an average age of 36.2 years. Three accidents, 2 suicides and 1 undetermined case comprised the subway-related deaths.

## Discussion

As China’s commerce, finance and science center, Shanghai developed extremely rapidly in the 2000s. This created thousands of jobs, attracting more and more migrants to study, work, and live in Shanghai. The migrants contributed to the population’s increase from 16 million to 23 million. Though the population in Shanghai was booming, the number of forensic cases in SPSB did not grow continuously, which fluctuated throughout the decade, with a minimum of 822 in 2009 and a maximum of 1170 in 2006. There were actually decreases in total, accidental fatalities and homicides through the latter half of this decade.

Traffic accidents were the largest proportion of the SPSB’s total forensic cases (45.0%, 4567/10135), which determined the trend in total SPSB forensic deaths. However, the number of traffic fatalities inspected by SPSB did not continue to increase throughout the decade; they began decreasing sharply beginning in 2004; laws and local regulations that took effect in 2004 contributed to this decrease. The local regulation encouraged independent third-party forensic science centers (not SPSB) such as university forensic medicine departments, private forensic science centers, and the Ministry of Justice Institute of Forensic Science to conduct the corpse inspections in noncriminal cases, especially traffic accidents. The Department of Forensic Medicine at Fudan University and the Ministry of Justice Institute of Forensic Science shared the responsibility for inspecting the SPSB’s traffic accident corpses so that the Bureau could concentrate on crime investigation. The true number of traffic fatalities in Shanghai was far more than 269 in 2009. But data from the independent forensic centers were unavailable for this study. Work-related injuries, the majority of non-traffic accidents, decreased rapidly and remained at a low level during the 2000s, although construction sites and industrial jobs increased in number. Awareness of workplace safety and protection increased based on lessons learned from past accidents.

Homicides were investigated entirely by the SPSB. The number of homicides did not increase along with the population; it remained stable with little fluctuation, the result of the SPSB’s crime control. The five leading causes of homicides were sharp force injuries, blunt force injuries, ligature and manual strangulation, smothering and drowning. The common causes of death in homicides in China are significantly different from those in Western countries, where firearms are the leading cause of homicides. A total of 23 victims in Shanghai were killed by firearms during the entire decade among a population of at least 16 million. This is extremely rare compared with data from the USA, which has a population of approximately 300 million; in that country, 31000 deaths were caused by firearms in 2007 alone [2]. Compared with the previous decade, gunshot victims decreased from 26 to 23, even with the booming population [3]. China’s strict gun control policy may be the reason for so few firearm injuries compared with the United States. The 7 syringe-injection cases were considered attacks intended to create an atmosphere of terror because Shanghai has great influence in China. The terrorists claimed that their syringes contained HIV-infected blood, and they injected innocent people in public places. Terrorist attacks do not solely include explosions on the London metro or attacks on the World Trade Center; chemical and biological terrorism have become realities in China. In addition to sarin gas and anthrax, HIV-infected blood can create fear as well. Chemical or biological weapons of mass destruction represent a new form terrorism. In response to the growing threat of terrorism with chemical and biological weapons, the city and nation should develop a comprehensive emergency health and medical services response policy [4–7].

The major cause of suicides was hanging in this decade, whereas ingesting drugs or chemicals was the leading cause in Shanghai in the 1990s [3]. Of the suicide overdose cases, carbon monoxide was the most common chemical, followed by pesticides, which were also the most common chemical used by suicide victims in Shanghai in the 1990s according to the data [3]. With the urbanization and industrial development of Shanghai in the 2000s, the agricultural population decreased, and thus, people’s exposure to pesticides decreased.

Of the 481 unnatural deaths in children, 278 were accidental deaths and 108 were homicides. The leading cause of childhood fatalities was traffic accidents. The leading cause childhood homicides was manual strangulation. Homicides were most commonly seen in children under two years of age, and female children were more likely to be the victims, which might reflect China’s preference for sons and the unfortunate result of female infanticide and discrimination in care practices for girls [8, 9].

Drug abuse deaths did not appear in the SPSB files until 1997 [3]. In the 1990s, 38 cases were recorded, nearly 4 times as many as those in the previous decade, and 1997 can be seen as the landmark year when drug abuse began to emerge in Shanghai. These decedents were markedly younger than the others (p<0.05). The drug abuse trend in Shanghai was similar to that in Hubei Province but earlier, and it began with sporadic cases [10]. The data from the 1990s confirm this conclusion.

Both illegal medical practice and subway-related deaths first appeared in the 2000s; there were no recorded cases of either cause of death prior to 2000. Illegal medical practice and subway-related victims were both significantly younger than all other decedents (p<0.05), and they were closely related to Shanghai’s development.

Illegal medical practice deaths are defined as homicides in Chinese; the people who perform these practices are not certified and have little medical knowledge. Many migrant workers in Shanghai are low-income with poor education, and they typically have no medical insurance [11–14]. When they need medical service, cost is a key factor in their decision-making. Illegal medical practices emerged against this background, being much cheaper than legal care. Additionally, some pregnant women who already have 2 or more children cannot obtain regular pregnancy checks at legal hospitals because of China’s family planning policy [15], which is one child per one couple. This policy also contributes to female infanticide [8, 15].

Shanghai’s subway system developed considerably from 2000 to 2009; running mileage increased from 63 km to 355 km, and annual passenger volume increased from 136 million to 2101 million; the subway-related deaths occurred against this background. For these deaths, witnesses are important in determining the manner of death. Subway deaths with witnesses in New York are more often suicides than accidents [16]. Detailed eyewitness descriptions of a decedent jumping into the path of a train or lying down on the tracks and suicide notes are compelling evidence of suicidal intent in most circumstances, although suicide notes are rarely found. Alcohol and drugs are risk factors in railway accidents [17, 18], but toxicology testing is not a routine procedure at the SPSB, and thus these data were unrecorded. Surveillance, education and public awareness should thus be enhanced to prevent subway-related deaths [19].

Regretfully, the SPSB has no routine toxicology test procedure. As a result, the alcohol and drug levels in most decedents were not recorded. Alcohol and drug testing is pivotal in determining manner of death in ambiguous cases such as drowning and injuries from jumping. Future routine toxicology testing, as well as investigations of decedents’ psychiatric states, is called for.

This study had some limitations. The fact that cause of death was unknown or unrecorded in so many cases is an issue, especially for the 91 homicides. The information in the archives was collected as is, even when it was unusual. Some of the SPSB MEs’ explanations included the following: (1) information was left blank or incomplete for unknown reasons; (2) bodies were incompletely dismembered and there was no clear cause of death; (3) a body was found in a suitcase immersed in water and cause of death was unclear because of decomposition; (4) the offender voluntarily surrendered to the SPSB, but the victim’s cause of death could not be certified through anatomic examination; (5) manner of death was determined based on the overall consideration of the case scene, an investigation and the suspect’s confession, but the cause of death could not be determined because of decomposition. Additionally, 10135 decedents was not the full number of cases examined from 2000 to 2009 in Shanghai, although they constituted the largest proportion of unnatural deaths in the city. A small number of unnatural deaths were examined by branches of the SPSB, and some traffic accidents were examined by independent third-party forensic centers based on jurisdiction. However, data from the SPSB branches and the independent forensic centers were unavailable for this study.

In conclusion, the causes of unnatural deaths in Shanghai, China, were significantly different from those in western countries. This retrospective study shows the characteristics of unnatural deaths in Shanghai and the relationship between unnatural deaths and the city’s development. Routine toxicology testing and more detailed background investigations are needed in the future. Additional research is also necessary for identifying the risks of unnatural deaths and improving public health by decreasing the number of these deaths.
